# Modern Smart Gadgets and Wearables for Diagnosis and Management of Stress, Wellness, and Anxiety: A Comprehensive Review

**DOI:** 10.3390/healthcare13040411

**Published:** 2025-02-14

**Authors:** Aman Jolly, Vikas Pandey, Manoj Sahni, Ernesto Leon-Castro, Luis A. Perez-Arellano

**Affiliations:** 1 Department of Computer Science and Engineering, Delhi Technological University, Delhi 110042, India; aman.jolly1994@gmail.com; 2 Electrical Engineering Department, Babu Banarasi Das University, Lucknow 226028, India; 04.pandey@gmail.com; 3 Department of Mathematics, Pandit Deendayal Energy University, Gandhinagar 382007, India; 4 Faculty of Economics and Administrative Sciences, Universidad Católica de la Santísima Concepción, Concepción 4070129, Chile; eleon@ucsc.cl; 5 Faculty of Psychology, Autonomous University of Sinaloa, Culiacan 80040, Mexico; luyz@uas.mx

**Keywords:** stress detection, smart gadgets, health sensors, anxiety, psychological behavioral

## Abstract

The increasing development of gadgets to evaluate stress, wellness, and anxiety has garnered significant attention in recent years. These technological advancements aim to expedite the identification and subsequent treatment of these prevalent conditions. This study endeavors to critically examine the latest smart gadgets and portable techniques utilized for diagnosing depression, stress, and emotional trauma while also exploring the underlying biochemical processes associated with their identification. Integrating various detectors within smartphones and smart bands enables continuous monitoring and recording of user activities. Given their widespread use, smartphones, smartwatches, and smart wristbands have become indispensable in our daily lives, prompting the exploration of their potential in stress detection and prevention. When individuals experience stress, their nervous system responds by releasing stress hormones, which can be easily identified and quantified by smartphones and smart bands. The study in this paper focused on the examination of anxiety and stress and consistently employed “heart rate variability” (HRV) characteristics for diagnostic purposes, with superior outcomes observed when HRV was combined with “electroencephalogram” (EEG) analysis. Recent research indicates that electrodermal activity (EDA) demonstrates remarkable precision in identifying anxiety. Comparisons with HRV, EDA, and breathing rate reveal that the mean heart rate employed by several commercial wearable products is less accurate in identifying anxiety and stress. This comprehensive review article provides an evidence-based evaluation of intelligent gadgets and wearable sensors, highlighting their potential to accurately assess stress, wellness, and anxiety. It also identifies areas for further research and development.

## 1. Introduction

**Figure 1:**
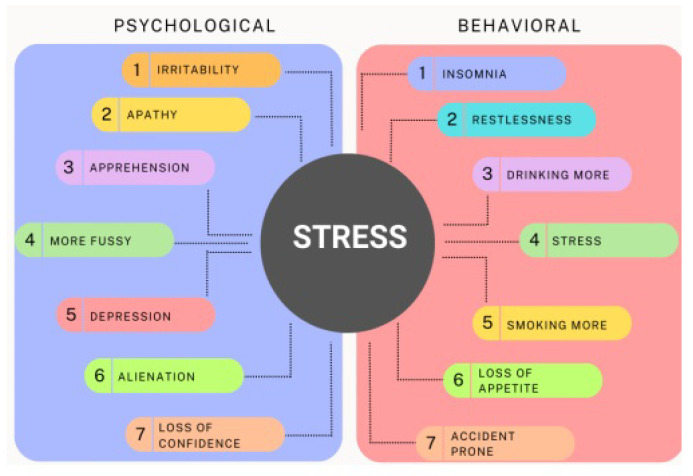
Psychological and Behavioral stress symptoms.

Many people across the globe, regardless of age, sexuality, or occupation, suffer from stress at some point in their lives. This pattern might be attributed to various factors, including changes in the working environment, rising workloads, shifting dietary habits, and new technology advancements. As a result, the importance of stress detection devices has grown significantly during the last several years. Stress is unavoidable, and it is vital to protect human resources throughout the globe from the rising effects of stress [1]. According to [2], people’s stress levels are rising as a result of contemporary living, where many factors contribute to stress, including the desire to succeed academically and professionally. Individuals who are in a stressful situation are more likely to suffer from a variety of psycho-social and physical health issues. Asthma, Rheumatoid arthritis, Anxiety disorders, Depression, Cardiovascular disease, Chronic pain, HIV/AIDS, Stroke, Cancer, and early death are all linked to the existence of stressful life and health issues. In addition to stress-related physiological disorders that can often cause long-term harm or destruction in humans, numerous psychological problems can lead to suicides. Stress can be mitigated by practices such as workouts [3], stress management strategies [4], restricting the signal strength of responding to emails [5], meditation techniques [6], listening to music [7], massage [8], and chewing gum and stress reduction [9]. As a consequence of this research, it was concluded that anxiety has profound effects on individuals, and many people’s lives may be improved by taking safeguards during the early stages of anxiety. This study is primarily focused on psychological and behavioral stress. Various symptoms of psychological and behavioral stress are illustrated in Figure 1.

**Figure 2:**
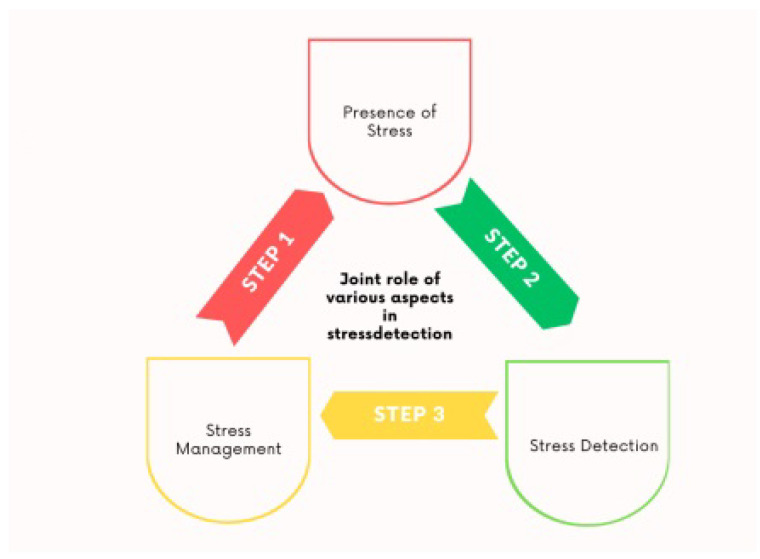
Joint role of various aspects in stress detection.

Anxiety can manifest as a temporary response, akin to transient emotional states, but it may also develop into a chronic condition, particularly under prolonged or excessive stress. Physiological processes play a critical role in shaping both emotional experiences and anxiety, with negative emotions often serving as symptoms of heightened or sustained stress levels. Both feelings-monitoring devices and stress-identification devices have been studied in this work, together with the emphasis on the numerous dimensions and roles of machine-learning algorithms due to their intrinsic interdependence. Smartphones and tablets are the most adaptable versions of computer technologies [10]. They are small, energy-efficient, and extremely computing-effective sophisticated gadgets that are carried by two out of every three individuals on the planet [11]. Throughout the years, several sensors have been incorporated into cell devices. It is now possible to gather actual information from several elements of human existence because of improved computer capabilities and novel sensors. Data-driven modeling is primarily concerned with gaining a better knowledge of how things function in the real world via experimentation and the usage of fast computing machines. As a result, research using cell phones has been conducted, ranging from human action detection to mental health recognition. Constantly rising personal information from omnipresent gadgets linked to activity, habits, and social interactions opens up a slew of new possibilities for improving social life and developing prediction and helping systems [12]. The many features of stress detection and stress management are shown in Figure 2.

As a result, early diagnosis and treatment of anxiety are critical for improving behavioral wellness and general wellbeing in the human population. Russo et al. [13] describe work-related anxiety as a detrimental psychological reaction that happens whenever the demands of a task do not meet the talents, expertise, or demands of the employee, and this may result in weak health and damage. Furthermore, work-related anxiety is associated with a rise in the incidence of casualties, tardiness, and lower performance. A stress-monitoring device may thus be advantageous for employees, learners, and anyone who is exposed to the challenging position that exists presently to self-manage their psychological wellness and wellbeing. According to [14], the following two qualities make the issue of anxiety measurement difficult and deserving of further investigation:

- Stress largely depends on the individual; what stimulates the anxiety response in one individual might not do the same in another.
- Stress identification has a tough time defining the facts of the matter. A stress event’s start time, length, and severity cannot be determined because of its subjective character and ongoing character.

There is no clear way to measure anxiety. Physiological, behavioral, and emotional responses all play a role in the body’s reaction to stress. Wearable technology may be used to track some of the physical reactions, such as a rise in heart rate or perspiration. The behavioral and emotional elements of the stress reaction, on the other hand, cannot be directly measured. The most common sign of stress is muscular strain, but sleeplessness is a close second in our 24/7 world. A lack of sleep, excessive alertness, or difficulty sleeping may all be symptoms of insomnia. Does someone’s intensity of anxiety influence the condition of someone’s sleeping, even when one sleeps well? It is really difficult not to acknowledge the abundance of commercials for sleeping prescriptions presently, implying a severe community healthcare crisis. Various investigations have established that obtaining a decent night’s sleep consistently is critical for decent wellness, but prolonged sleeplessness is frequently linked to a variety of mental issues. Insomnia is assumed to be caused mostly by emotional stress. The outcome is an agitated mental state in which ideas run about, ricocheting from neuron to functioning brain, never pausing, much less enabling the individual to fall asleep [15]. How long rest do individuals need to feel refreshed? Generally, 8 h of rest is recommended, while some individuals may manage with as little as 6 h of rest and still feel refreshed. Others may need up to ten hours. According to new research, teenagers, defined as those aged 12 to 22, require more than 8 h of rest. Hormone shifts, high coffee consumption, less or no workout, extreme thirst, circadian rhythm abnormalities, rotating shifts, liver toxicity, and several other daily habits all detract from one’s sleep habits and may influence sleeping patterns [16]. A slew of the latest gadgets claims to be able to evaluate PA in innovative and better ways. Such gadgets create human metrics based on sensor outcomes using a variety of sensors and algorithms. Pedometers are used in conventional foot trackers to determine regular foot counts. Pedometers are not as precise as accelerometer sensors, which are the current version for gathering PA information [17] while being cheaper and more economical. An accelerometer is found in all current activity tracking and smartwatches. For certain metrics, these gadgets are regarded as less precise than research instruments [18]. These are, nevertheless, less intrusive, less expensive, have greater configuration, are more user-friendly, and are widely employed in the investigation. Many accelerometer-based workout trackers monitor acceleration in three different directions [19] and may be employed to evaluate mobility type, record footsteps, compute calorie consumption and energy intensity, and predict sleeping habits, among other things. These measures have varying degrees of reliability and validity. In a review published in 2015, Evenson et al. [20] reported good validity for steps but poor validity for “Energy Expenditure” EE and sleep. Furthermore, they discovered that some devices had high reliability for steps, distance, EE, and sleep. Gyro sensors, magnetometers, pressure sensors, and altimeters are also included in certain wearables [21,22,23,24,25]. By monitoring gravity acceleration, alignment, and angular speed, a gyro might improve gadget precision and accurately predict which movement an individual is undertaking. A magnetometer is a digital compass that may increase motion detection accuracy by sensing the gadget’s alignment concerning Earth’s axis. Magnetometers increase stability by accounting for gyro drift, an issue with gyros in which the rotational axis wanders away from the real motion over time and must be corrected regularly. Inertial measuring units are frequently made up of motion sensors, gyroscopes, and magnetometers (IMU). IMUs are used to compute positions in most cell phones, and a growing share of health trackers use these units to provide more precise measurements. Barometers or altimeters monitor fluctuations in elevation and may be used to enhance certain measures (e.g., EE) as well as provide other metrics (e.g., ascended floors). In the world of gadgets, photoplethysmography (PPG) is a fairly recent method. PPG [24] is an optical approach for estimating HR by measuring variations in blood flow under the skin. Light is projected onto the skin via a light-emitting diode, which is changed by the HR and retransmitted to the detector. However, signal noise is caused by mobility, ambient light, and tissue compression. Therefore, cleaning techniques often incorporate acceleration information to improve HR estimates. Because there was a considerable indication that gyroscopes may help minimize PPG signal distortion, we are expecting to see more products featuring PPG detectors in the upcoming. Many gadgets feature a built-in global positioning system (GPS) detector to supplement PA data collection. This is particularly suitable for large fitness trackers and athletic wearables designed for those who are physically engaged. More data, such as location, velocity, and elevation, may be tracked with a GPS [25]. There seem to be many commonalities between various sorts of gadgets, and categorizing them may be challenging. In this work, we will refer to wrist-worn devices that can monitor and communicate PA information with a cell gadget as “wearables”. A smartwatch is a wrist-worn gadget that, in most cases, works as an addition to a smartphone, displaying alerts and tracking PA and other parameters. Newer smartwatches frequently entail a touchscreen interface and may handle sophisticated functions such as large fitness patterns. Activity trackers (also known as smart bands or fitness bands) are gadgets that are designed to monitor PA. They are often put on the wrist or hip. An activity tracker is frequently less costly than a wristwatch due to lower-cost electronics and fewer sensors. As a consequence, it has a longer battery life and a more restricted user interface for showing tracked data [26]. Portable devices have become more common as a result of recent technological advancements. Though consumers are most familiar with wrist wearables (thanks to hand watches), and this is one of the least conspicuous locations, A wrist device is picked for our trials [27]. Furthermore, owing to the hands’ action, the wrist is subjected to extensive mobility, which adds noise to the data and further complicates the already difficult challenge of stress identification [28,29]. Other titles, such as athletics wristwatches and GPS devices, are also employed to describe a hybrid of wearables and activity trackers. Some gadgets include a clockwork and analog display with electronic sensors, making them hybrids [30]. The majority of the time, one needs a smartphone plugin to obtain the information needed; nevertheless, proper step counting may be shown directly on the watch display. Wrist-worn sensor systems, techniques for intelligently analyzing data from wrist-worn gadgets, and techniques for displaying observed information are all covered in this thorough overview. The most important elements of commercialized wrist-worn gadgets were reviewed. Then a topology of the different types of noninvasive wrist wrist-worn detectors, functions, and methods was found and mentioned in depth in the next subsections. Some problems that need to be addressed (such as detector consistency, information homogenization, on-device intelligence, and the absence of smart assessment visualization techniques) were also discovered [30]. The objective of conducting the review is to understand the process of recognizing stress, monitoring wellbeing using wearable sensors, and understanding the techniques and components of modern health monitoring devices and how these sensors can be utilized for stress-monitoring mechanisms. The focus of this paper is on wearable devices, in which unobtrusive monitoring of health can be done. The review has taken the research work of unobtrusive stress and wellbeing monitoring and not just laboratory-simulated experiments. Adding useful portable and wearable devices with precise algorithms that can do apt analytics of data and calculations that can give a valuable device to the checking and the executives of mental issues. The breakdown of the paper’s architecture is as follows. Research topics and taxonomy are presented in Section 2, as well as the methods utilized to conduct the review. A review of stress detection criteria, everyday stress effects, and commercialized wrist-wearable measurement device characteristics can be found in Section 3. Section 4 provides a thorough breakdown of the many methods for detecting stress. Data visualization and smart data processing are the focus of Section 5. Section 6 discusses the current work’s limitations and potential scope. Section 7 concludes with a look forward to the future.

## 2. Methodology

The technique described in this SR has been based on the “Preferred Reporting Items for Systematic Reviews and Meta-Analyses” (PRISMA) declarations. PRISMA is a minimal collection of systematic reviews and meta-analyses that is related to evidence. It is usually intended for reporting reviews that assess the impacts of interventions, though it can also be utilized to record systematic reviews with objectives other than assessing interventions, for example, prevalence, diagnosis, or prognosis.

### 2.1. Search Strategy

As a part of this work, a systematic literature review (SLR) focusing on stress detection in the human body is being conducted to assist us in better understanding how to overcome various stress-related diseases. The initial information sources consist of four primary academic literature collections: Science Direct, IEEE, Springer, and Scopus databases. When researching a certain research issue or topic area, researchers conduct systematic literature reviews (SLRs) to acquire information on relevant research in that field. The last search was on 20 December 2023 and repeated database searches were conducted using keywords to locate significant academic material. The essential phrases were searched in the topic and title, as well as the topic, title, and abstract, with no time restriction (Scopus and others). An article, review paper, proceedings paper, bibliography, and article were the only document types allowed. Table 1 lists the terms that were utilized in Scopus. Furthermore, other keyword spellings were looked for which totaled 15 records.

#### The Brand Selected for Research

On 15 December 2021, we checked PubMed, Embase, and Ovid MEDLINE to see how frequently the most significant brands had been used in prior research. We used an important word search with no restrictions for every request. The results were separated into two categories: validating and reliability investigations and information-collecting investigations. We conducted two groups of inquiries to determine what brand was the most appropriate. We developed a product keyword search for companies that are either (1) one of the top five bestselling brands in 2019 and 2020 or (2) have launched 10 or more distinct gadgets during the first batch. We then filtered the titles, descriptions, and methodology parts of the papers from the resultant list. Such identification was performed to (1) eliminate publications that were not relevant to the study and (2) determine other brands that were employed in these trials. Then, we made a list of such brands and did another batch of inquiries, one per newly discovered brand. Eventually, ten names were added. In the results section, where we summarize the observations, we include the particular search term utilized for every brand.

### 2.2. Scrutinizing of Paper for Study

**Figure 3:**
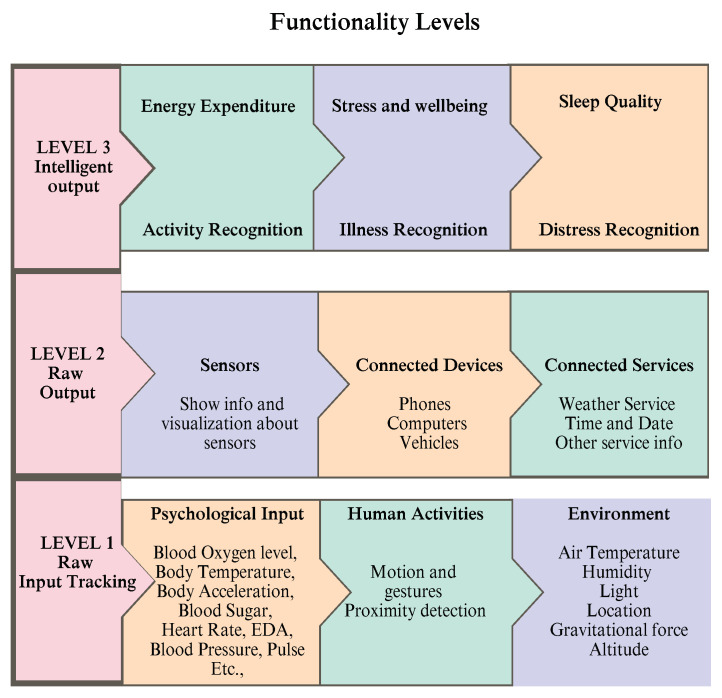
The three-tiered classification system for wrist-worn gadget features.

The primary studies (PS) selection procedure is divided into four stages: detection, admissibility, inclusion, and multiple screening. The initial stage involves identifying each potentially important study. There were 6407 results in the first search. A systematic examination of several databases and sources, including full-text articles, “Science Direct”, Springer, Scopus, and “IEEE Xplore”, yielded conference proceedings. To remove multiple copies, all results were screened and analyzed, yielding a total of 195 studies, 28 percent of which were focused on emotion stress detection, 17 percent on scientific viability and consistency, as well as 33 percent were investigatory research on science-based recent applications and innovations. The second stage does a preliminary evaluation by screening titles, keywords, and abstracts. At this point, 6540 records have been excluded because it does not fit the inclusion requirements, particularly in terms of the scope of research and optimization topic. These two records marked as comprising and those marked as unclear were forwarded for additional review. According to Figure 3, an SR database assessment is shown. It was also necessary to go through by hand the bibliography of all pertinent publications and review papers. The rest of the documents were thoroughly examined. The additional data and abstracts of the papers were analyzed to determine which research should be included and excluded in the present systematic review, and the following criteria were used as discussed in Table 2. The inclusion and exclusion criteria for the systematic review were established to ensure a robust selection of studies. The inclusion criteria stipulated that papers must be peer-reviewed (I1), written in the English language (I2), and have no specific publication timeframe limitations (I3). Additionally, included papers were required to be published research articles or full-article publications (I4), while the standard of the paper was considered blind to its impact factor (I5). In contrast, the exclusion criteria ruled out papers that did not focus on body stress-related studies (E1), grey literature (E2), duplicate research and publications (E3), and non-peer-reviewed formats such as Ph.D. theses, working papers, and project deliverables (E4).

### 2.3. Objectives of Current Study

The purpose of this research is to use behavioral available data, psychological available data, and advancements in technologies to assess and enhance individual psychological health issues. Its concentration is on cell phone statistics, then particularly concerned with predicting human psychological satisfaction, which necessitates reaction information that will be anticipated in upcoming scenarios. A wide scope of gadgets involving an assortment of sensors has been created to catch the physiological and social information and make an interpretation of them into aggregates and states connected with psychological wellbeing. Such frameworks intend to recognize practices that are the outcome of a basic physiological modification, and thus, the crude sensor information is caught and changed over into highlights that are utilized to characterize conduct markers, frequently through AI. Nonetheless, because of the intricacy of passive information, these connections are not straightforward and should be comprehended. Moreover, intrapersonal and relational contrasts should be viewed when deciphering the information. Additional information and computational approaches are examined concerning the issue. A further goal of this systematic review (SR) is the development of an open-source knowledge platform to support future research on this topic by collecting and analyzing key findings from previous research, summarizing and comparing them, and identifying the issues and limitations that have arisen as a result of this work. Research on wrist-worn detectors and gadgets was undertaken by assessing the current level of information in this field. The three main investigation questions were formulated during the designing phase of the study, and they are discussed and evaluated throughout the article. The following are the investigation questions:

RQ 1: How sensors and wrist-wearable devices are useful in stress detection?
RQ 2: What are the essential factors for creating a concept for wearable electronic gadgets?
RQ 3: What are the Limitations and Research gaps in existing systems?

- To examine sensors used in wrist-worn gadgets. The literature study found three categories: environmental sensing, physiological sensing, and activity sensing.
- Reviewing approaches for the efficient processing of information from wearable detectors and gadgets.
- In-depth examination of approaches for visualizing statistics obtained from wearable sensors and devices.
- To compare and contrast the most popular commercial wrist-worn gadgets on the market.
- To create a taxonomy of wrist-worn device sensors, functions, and techniques.

To achieve these objectives, a thorough literature study was conducted initially. “Sage”, “Emerald”, “Google Scholar”, “MDPI”, “Science Direct”, “IEEE Xplore”, and “SpringerLink” were used to explore the citation indexing databases and Internet of publications to locate related papers published in the past ten years. In addition, an Internet search was conducted to find the top wrist-wearable gadget makers. Documents such as white papers and manuals from manufacturers and peer-reviewed research studies were consulted, and the results were analyzed.

#### Taxonomy

There has been no clear characterization of the nomenclature of these gadgets in terms of capability via this systemic evaluation of intelligent wrist-wearable gadgets. Depending on their functions indicated in Figure 3, a taxonomic classification of wrist-wearable smart bands and smartphones has been established. In the classification of smart wearable technology, there are three stages of capability, and a smart band on a tier higher than the first may contain certain features from lower tiers of capabilities. At Level 3, wrist-worn smart bands and smartphones are the subjects of this article. The taxonomy’s first level is made up entirely of raw input monitors. Everything from HR and blood pressure to temperature and moisture to sound and light to movement and speed is included here. One of the main differences between Level 1 capability wearable bands and greater capability wearable bands is that they do not exchange any knowledge about their wearer directly with the raw inputs from the wearer’s physiology, activities, or surroundings. Raw input monitoring and raw output indications are included in Level 2 functionality wearables. Graphical interfaces such as LCD, e-ink, or another sort of screen, audio, vibrations, lighting with monochromatic or multiple colors and patterns of flashing, electromechanical indications, or other graphical, auditory, or indications may all be used to communicate with a device. A sports monitor is a good example of a smart band with Level 2 capabilities since it evaluates the user’s physiologic inputs and shows the resulting value. In certain cases, smart bands and smartphones may also display raw data from a distant source. Despite the lack of an input data supervisory authority, these devices are nonetheless classified as Level 2. Level 3 of the wearable bands and smartphones capability hierarchy comprises gadgets that provide some meaningful knowledge to the wearer. There are several ways in which Level 3 wearable may present additional information generated from the inputs, unlike Level 2 devices that merely display raw numbers. Fitness trackers, wearables, and other wearable safety gear are examples of these gadgets. Using well-known mathematical formulas, principles, stats, and even machine-learning methods, they turn raw inputs into useful outputs. The typology does not need the smart output computation to be performed on the wearable device itself; it may be performed remotely in the cloud or on other connected devices instead of smartphones or computers. When it comes to Level 3 wearable, the basic inputs may be transformed into fresh information, or the basic inputs can be gathered externally and shown for the user to see. Figure 3 depicts a three-tiered hierarchy of wrist-worn devices’ functions. The current focus of SR is on wrist-worn devices with smart output at Level 3.

## 3. Stress

Stress or anxiety is a commonly utilized word; however, it is not easy to agree on stress’ meaning since stress is a personal process that is difficult to describe phenomena [31]. Furthermore, if researchers cannot identify anxiety, how might they measure stress? “Merriam-Webster Dictionary” describes anxiety as an emotional, physical, chemical, or component that generates physiological and behavioral strain, and it might be an element in illness. Non-formally, anxiety might be described as the person’s reaction to any stressful or dangerous scenario. The brain triggers the anxiety symptoms in reaction to physical records from the nose, ear, and eyes, canals. Whenever a person perceives danger, it might be genuine or imaginary, defense systems of the central nervous system launch a quick, adaptive activity described as the “defense” response or the stress response to defend itself. The hypothalamus receives instant feedback from the central nervous system indicating danger. The hypothalamus is equivalent to the control center of the nervous system [32]. The hypothalamus governs involuntary bodily activities through the “autonomic nervous system” (ANS). ANS comprises two distinct parts “sympathetic nervous system” (SNS) and the “parasympathetic nervous system” (PNS). When it comes to SNS, one may compare it to the gas pedal on an automobile. In in-flight scenarios, after obtaining the danger indication, the hypothalamus triggers SNS, and when the SNS is activated, anxiety chemicals like adrenaline and cortisol are released into the body. When the sympathetic nervous system (SNS) is engaged, the HR increases, muscles tense, blood pressure rises, and the rate of breathing rises. Volumes of the airways in the lungs rise. Sensitivity improves when brain oxygen levels rise, according to Zaneveld et al. [33]. Additionally, the rise in blood sugar elevates a person’s level of energy. The above-mentioned adjustments boost the ability and muscular strength, narrow the response period, and enhance attention. If this system operates as planned, it assists a person to remain concentrated and attentive. Whenever the threat has passed, the stress response is reduced by the PNS, which is like the braking in a vehicle. Physiological stress is the term used to describe a person’s physiological reactivity to some kind of stressor. Perceived stress is a different kind of stress that results from a person’s subjective assessment and perception of a difficult circumstance. Perceived stress may be quantified using monthly self-reports obtained from the participants [34]. In other words, it is the assessment of anxiety from a person’s point of perspective. Ideally, experts assume that the two anxiety tiers have consistency. Furthermore, the opinion could be prejudiced. This could fluctuate individually present instances relating to the uniqueness of the person [35]. They revealed that while physiological information is identical for both males and females in their experimental trials, females tend to indicate anxiety levels greater than men on self-reported information. Stress level also has the drawback of missing instances of stress since self-reports are only taken at certain intervals. Stress has a detrimental influence on memory, and individuals have a propensity to experience unpleasant situations, particularly if the data-collection time is extended. Following these difficulties could cause disparities between the recorded cognitive anxiety concentrations and subjective anxiety intensity. The gathering of felt stress statistics is also much more complicated than physiological knowledge gathering. However, for the gathering of the physiological parameters, an inconspicuous portable technology might be adequate for the felt stress. Volunteers need to complete certain questionnaires regularly, which could put a strain on the person. When a person is under stress, they are more likely to withstand perilous situations. Up to a certain degree, tension may also be good in stressful circumstances such as a presentation at the workplace or a test at education [36,37]. As stated, anxiety is a phenomenon of the body that supports and saves in crucial instances. Once a person’s stress level has risen over a particular point, stress ceases to be useful and begins to harm their physical and mental wellbeing, performance, and overall happiness. The main factor behind this is that our nervous mechanism is unable to distinguish between different physical and emotional hazards. In emotive demanding situations including a disagreement with a partner, the neurological mechanism reacts in the similar way it would in a life-or-death situation. If this engagement occurs often and a person experiences increased stress, their body will be under constant stress, which may lead to major health issues.

## 4. Stress Detection Techniques and Sensors

McGrath identified two basic types of environmental factors: interior and exterior. The former is more likely to be used in the house or office and, as a result, measures things like temperatures, moisture, daylight, quality of air, sound, and gases. The latter includes, among other things, information on air quality, quality of water, traffic noise, and climate. According to Willox et al. (2015) [38], climate change may harm mental health among Circumpolar Indigenous groups. Therefore, efforts to address this issue via research, policy, and mental health programming should be a top priority. For Cianconi et al. (2010) [39], rising temperatures and heat waves may induce physical and mental illnesses in humans, as can droughts or fires; forest and glacier loss; the disappearance of rivers; deserts; and a host of other natural disasters. This section focuses on some of the sensing sensors that are currently in use. This section provides a snapshot of environmental detectors that may be used to assess stress levels. Due to their direct or indirect impact on things, environmental conditions might provide difficulty in precise detection. If one wants to minimize these factors, it is best to know as much as feasible about the status of one’s natural surroundings so one can figure out what the consequences will be [40].

### 4.1. Air Temperature

The temperature is represented by the reflected rotational, vibrational, and translational motion of physical matter. Thermal sensors may be classified into four broad categories: Negative temperature coefficient thermistors, resistance temperature detectors (RTDs), thermocouples, and semiconductor-based detectors [41]. As per Aarnes et al. (2017) [42], skin surface warming of patients not only improves thermal comfort but has been shown to reduce anxiety in a pre-hospital setting. An NTC sensor has a temperature-sensitive resistance that is very precise. RTD is focused on the temperature-dependent resistance of the RTD component and may be either a sheet or a wire encased in ceramics or silicon. Sensors that use thermocouples, as their name indicates, are made of two wires of dissimilar materials, and the variable voltage depicts temperature variations. Two similar diodes with temperature-sensitive voltages are the basis of semiconductor-based sensors [43], which are mounted on integrated circuits. Polyimide foil has also been used to include capacitive and resistive temperature sensors [44]. Predicted Mean Vote (PMV) is a portion of an ISO standard that tries to forecast the average thermal feeling of a mass, which is the perception of temperatures between hot and cold [45]. The combination of air temperature and skin temperature may be effective in determining the thermal feeling of a person [46]. There are drawbacks to using any of the above-described kinds of thermal sensors. Because of their exponential nature, NTCs perform best at low temperatures, and their output must be linearized. A platinum RTD is preferable to a nickel or copper RTD because platinum is more stable and reproducible, even though platinum is more costly. Detectors that use thermocouples and semiconductors are inaccurate and sluggish to respond, respectively [47].

### 4.2. Altitude

“Barometric pressure sensors” are mostly used to figure out altitude [48]. Depending on barometric elevation data, embedding systems (e.g., cell phones) may also employ pressure-regulating sensors for lateral positioning identification systems. Asirdizer et al. (2018) [49] found that high altitudes above 1500 m, winter median temperatures lower than 10 C, and hard temperature changes above 25 C between winter and summer of settlements were important factors that affected female suicide rates appropriate to knowledge which defined in previous studies. It has multiple outcomes: pressure and temperature values, which, when combined, are used to calculate altitudes [50]. The effectiveness of navigation systems may be improved by integrating instrument readings with GPS knowledge. When participating in sports like rock climbing or scuba diving, it is a good idea to keep track of altitude measurements. Because pressure-regulating sensors lose stability with age, the most typical issues they face are instability and inaccuracy issues. As piezo-resistive pressure gauges degrade with time, so does the accuracy of the readings. In addition to this, pressure gauges must be able to breathe in dry, humid, and wet climates, which might be difficult. When atmospheric pressure changes, it may lead to erroneous pressure readings, which can be fixed using dynamic pressure measures [30,51,52].

### 4.3. Light Sensing

The luminance of a smartphone screen is usually adjusted to match the ambient sunshine level, thanks to an illuminating detector [53]. There is a strong correlation between exposure to naturalistic light during admission and improved mental health in rehabilitation patients, as shown by West et al. (2019) [54]. Instruments that transform daylight into voltage or current are primarily silicon PIN-type silicon photodiodes [55]. As far as known, they can pick up wavelengths between 400 and 1100 nanometers (nm). While passing through the depleting zone of a diode, light may strike an atom, liberating electrons and causing an electrical current between the cathode and anode. Photodetectors and light-dependent transistors are additional forms of photodiodes. Ambient UV or fluorescent light detection in wrist-worn devices, such as the Fitbit Surge or Microsoft Band, is also present in these devices [56,57,58].

### 4.4. Ultraviolet (UV) Light

The ultraviolet radiation (UV) wavelength is known for causing skin disorders ranging from wrinkling to skin cancer that ultimately leads to the degradation of mental health and an increase in anxiety levels. Particular optical detectors may be used to measure UV radiation on the wristband (My UV Patch [59]) and wristbands (Microsoft Band [58]). As a safety measure, UV sensor-powered smart gadgets may be employed. When it comes to UV sensors, they are part of the light-sensing group, which means that each of them can detect a certain spectral area. Detectors based on the concept of photoconductivity assess variations in the light intensity emitted or even changes in light beams in response to any additional interaction [59,60,61].

### 4.5. Humidity

According to Wolkoff (2018) [62], several immobile sensors evaluate the quality of the air on a particular scale since moisture is also a factor in pollution levels. Humidity level plays a vital role in determining the anxiety level and mental condition. The Sens-Podfrom Sensors, in addition to moisture, include detecting abilities for a variety of gases and noise as well as temperature may also be used to do similar humidity measurements. A wrist-worn device may also be used to collect humidity data on the fly. Fiber-based technologies may be used for moisture monitoring [63]. Specific luminous systems include dyes, fiber-coating polyimides, and thin, film-coated tin dioxide and titanium dioxide fibers coated with moisture-absorbing polyimides as detectors. These detectors also have various issues that, if not solved, might provide inaccurate results. Non-linear responses are caused by a broad variety of temperature and humidity conditions, as well as by imperfect observations [64].

### 4.6. Sound

Many smart gadgets, such as cellphones, employ audio detectors, as do specialized gadgets, such as the Angelcare newborn sound and movement sensor device [65]. Lan et al. (2020) [66] support the hypothesis of an association between traffic noise and more severe anxiety. A wrist-worn device with a noise sensor and recording option might be used to study a person’s stress level and its association with ambient noise levels (e.g., noise pollution in urban areas) or simply by measuring the pitch level [67]. According to Kamišalic et al. (2018) [30], a model for a portable gadget has been evaluated. Audio waves may cause changes in pressure, which can be detected by piezo-electric sensors, allowing for more accurate decibel measurements [68].

### 4.7. Gravitational Force

The acceleration of the item, expressed as a speed of variation, is what is referred to as the gravitational attraction or G-force. “Accelerometers”, which are electromagnetic gadgets, are employed to assess both stationary and kinetic loads, such as gravitation or vibration and mobility [69]. However, the gravitational force measured individually does not determine the anxiety. The inputs from the g sensor, along with other sensor data, can identify the anxiety level and mental health conditions. Accelerate is commonly measured using a three-axis instrument. As acceleration forces occur, the capacitance between the plates of the accelerometer varies, and this capacitance is used to calculate the acceleration. Material that generates electricity via piezoelectricity has been employed in several applications [70]. When subjected to acceleration, even the smallest crystal formations produce an electrical charge. The most prevalent use of this kind of detecting is in aircraft, but it has recently been employed in athletics as an influence detector in football and winter sporting events as well. The G-force sensor might be used as a wrist-worn device, detecting by concentrating on the oscillations [71]. Table 3 shows a list of environmental conditions detecting sensors available in smartphones, smart bands, and smartwatches.

## 5. Stress Assessment

This section explains the technologies that are used to detect stress-related variables. The wrist may be used to collect a plethora of physiologic and behavioral information, including weather, HR, physical movement, and social media activity, among other things [72]. In medical practice, a personality scale may be used to quantify stress. Rather than a participant filling out a questionnaire, the VAS delivers a swift quantitative evaluation in a 10-point category [73]. Since stress is characterized as a situation in which equilibrium is endangered, the adaptation mechanisms that are engaged would produce both behavioral and physiological alterations. To grasp this biological stress mechanism, much research has been undertaken examining the behavioral and physiological alterations in the person during stress generation tests. The study of physiological or pathophysiological changes in response to stress is crucial to the generation of innovative pharmacological medicines for reducing stress [74]. In the fast growth of contemporary society, individuals are coping with stress and work exhaustion regularly; consequently, like pain evaluation, stress evaluation might also benefit from monitoring throughout everyday activity. Without further generating stress to the individuals, such situations are correlated with weariness and work-related stress to address the issue of workers’ emotional and physical wellbeing [75].

### 5.1. Physical Stress Assessment and Sensors

The relationship between pupil stress and sickness is well-documented, and it is especially prevalent among university graduates. Psychological discomfort among college students was shown to be much greater than among the general public college kids who are especially vulnerable to anxiety; however, there is a definite correlation between pupil anxiety and sickness [76,77]. Some diverse behavioral markers that are useful in the evaluation of stress are listed below:

#### 5.1.1. Heart Activity

Considering stress induces basic abnormalities in the “autonomous nervous system” (ANS) that have large impacts on “heart activity” (HA), several helpful identification approaches for anxiety are focused on heart-related information [40,78]. The soundness of one’s cardiovascular system is closely correlated with one’s heart rate, which is an extremely important health measure. The heart rate is the number of times the heart beats per minute, which represents several physiological states, such as biological workload, tension at work, attention to tasks, sleepiness, and the active state of the autonomic nervous system [79]. Heart activity might be characterized by an “electrocardiogram” (ECG), which would be obtained by monitoring the electric impulses of heartbeats. Much research on HA is devoted to 3 elements of the cardiac: time intervals, frequency field, and “non-linear” properties of HA. A study in the “time domain” concentrates on factors including HR [80,81]. According to Khan et al. (2016) [72], the pulse is the rhythm of cardiovascular oscillations, which is indicated in beats/minute. ”Photoplethysmography” (PPG) is the most widely used technology for measuring heart rate. It is used in the detection of volumetric alterations in the circulation of blood in the periphery circulatory system. Utilizing electro-optic and mechanical detectors, the pulse information from the PPG may be acquired on the wrist and shown on a display. Hwang and Lee (2017) [82] describe how the optical sensing approach may be used to measure blood flow rates by measuring the intensity of light reflecting from skins using LED and photodiodes. Zheng et al. (2014) [83] demonstrated that an illumination system is employed to illuminate the vasculature, and a photodiode is utilized together, which is returned from or transmitted through the muscle. The intensity of transmitted light or reflecting by and from the skin, whereas the change in tissue relies on the amount of blood flowing through it. Within each cardiac cycle, the signal acquired with a photodiode indicates the pulsatile blood volume variations of the peripheral microvasculature, which are caused by a pressure pulse [84]. Although it is possible to generate a reasonably decent signal from transmitted light, the measuring site is restricted to those regions of the body where the signal may be recognized quickly and easily. This is not an option for a gadget that is worn on the wrist. An alternative measuring method is to employ a reflecting signal, which may be applied to several measurement locations, including the wrist. Although it has another drawback in that it is impacted by motion artifacts, pressure disturbances, and skin pigmentation, it does have certain advantages. Signal processing methods may be used to increase the accuracy of the heart rate (HR) measured using PPG sensing from the wrist [85]. It was shown by Fallet et al. [86] that a normalized least-mean-squares technique could be utilized to debilitate movement artifacts and to rebuild numerous PPG outputs from various combinations of injured PPG waveforms and information collected from the accelerometer. Adaptable band-pass filtering was employed to monitor the HR of the rebuilt PPG pulses. Nowak et al. [87] in 2015 described techniques for tracking heart rate via PPG data. The approaches that have been established are resistant to mobility artifacts. They employed spectrum reduction or “non-negative matrix factorization” (NMF) for data augmentation and movement artifact elimination. Zhu et al. [88] presented the MICROST model, which employs a hybrid method to assess human resource expenditure. There are three main components: acceleration categorization, initial frame analysis, and heuristics monitoring. Researchers at the University of Tokyo have designed and constructed a wristband-type PPG pulse rates monitor that is capable of avoiding mobility artifacts in everyday activities and identifying HRV. Ishikawa et al., in 2017 [89], using this technique, were able to drastically reduce motion artifacts generated by hand, fingers, and wrist motions. HR might be detected instantly and noise-resistant using pulse patterns with noise reduction depending on peak identification and autocorrelation. An individual’s heart rate was determined by Fukushima et al. [90] by comparing the amplitude spectrum of PPG and speed. A frequency-multiplexed sensing element was constructed by Lee and his colleagues utilizing complementary split-ring resonators [91]. These wristbands can spatially resolve wrist movement concerning sensor placement. The artery’s mechanical movement is detected by the disruption of the electric fields emitted by the source resonator. To validate the biological knowledge utilized to modulate the transmission, the observed perturbations are employed. To acquire a more precise reading of the pulse, the wristbands utilize both location recognition and pulse monitoring. Wearing a FitBeat [92] wristband, users can accurately measure their heart rate during strenuous activity. Additionally, it uses basic spectrum analysis methods to eliminate numerous causes of inaccuracy. Enhancing common filters and spectrum analysis tools reduces the computing burden dramatically. Skin’s optical qualities have been addressed in a sensor module design presented by Mohapatra et al. [93]. To obtain the best possible signal quality, they used an optical system with a 590 nm (yellow-orange) wavelength. Blood Volume Pulse may also be used to calculate HR [14]. The HR may also be measured using multi-channel WPPG detector data and accelerometer signals. Monitoring and calculating the HR may be done using infrared technology. Using infrared light, infrared wireless transfers data. Commercially accessible infrared sensors are employed in combination with Bluetooth technology for connectivity, according to [47]. Heartbeat observations may also be obtained utilizing an ultrasensitive pulse-driven detector constructed [94].

#### 5.1.2. Skin Temp

Both the central and periphery body temperatures are affected by stress. Although the inner average temperature rises in stress regard, the temperature of the peripheral skin regions drops. Rapid anxiety promotes peripheral vascular constriction, resulting in a fast reduction in body temperature [95,96]. The assessment of location affects the body’s temperatures. Skin temperatures may be assessed using infrared thermopiles [14], temperature sensors, thermoelectric impacts, or optical methods. The resistance of a thermistor fluctuates with temperature, which is why it is often found in wearable sensors [72]. Strain is a factor in thermometer performance. The thermistor strain impact is difficult to separate from the temperature influence. Another key difficulty is to appropriately assess body temperature. Wearable wrist devices monitor skin temperature, which is a few degrees lower than the body’s core temperature. In addition, skin temperature exhibits oscillations based on external temperature, while body temperature changes less than 1 C throughout the day [72]. Sweat evaporation and the sensor’s poor thermal contact with the skin are another issue since there is a diminished correlation between the skin’s temperature and the rest of the body. There is research that shows that the two may be linked. Using skin temperature wrist measurement with additional domain-specific information, Seoane et al. [97] provide an estimate of the body temperature. Anxiety and feelings may be measured using a combination of skin temperature and other metrics. It was shown by Sim et al. [98] that the skin temperature of the wrist may be used to assess a person’s subjective thermal feeling.

#### 5.1.3. Blood Volume Pulse

Blood volume refers to the quantity of blood in a given length of time in a certain area of blood tissue. Light reflected by the skin is measured by the blood volume pulse (BVP). Giannakakis et al. (2019) [99] conduct a comprehensive assessment of the BVP patterns that occur in stressful situations and provide sound practical advice for improving the efficiency with which stress may be detected. BVP measurements might be obtained by the use of photoplethysmography (PPG), a technique that uses light to detect variations in blood flow volume and provides information on pulse rate [86]. The blood volume pulse may be used to calculate HR and HR Variability [14]. Anxiety and heart disease may be detected using HR variability [100].

#### 5.1.4. Blood Oxygen Saturation

Excessive production of reactive oxygen species (ROS) or failure of the antioxidant system may both lead to oxidative stress. With its high oxygen consumption and many peroxidation-prone lipid cells, the brain is particularly sensitive to the effects of ROS. As defined by Khan et al. (2016) [72], oxygen concentration or oxygen levels is defined as the ratio of “oxyhemoglobin” in the blood to both “oxy” and “deoxy” “hemoglobin” in the body. When two LEDs, each emitting light at a particular wavelength, flash through or onto the detecting spot, the measurements are made [101]. The light intensity reflected through or from the body and skin is determined by the amount of hemoglobin and the amount of oxygen composition in the blood. This reflected light is collected by a light sensor. Oxygen-bound hemoglobin has a lower absorptivity than unbound hemoglobin. Thus, the wavelength of reflections allows optical analyses to be made. From the photosensor signal at every wavelength and the existing molecular absorptivity of oxygenation and de-oxygenated at every wavelength, the oxygenation is calculated [102]. The most typical difficulties that limit the precision of observations are kinematic artifacts and environmental wavelength interference. Problems like these can be solved by putting the detector closer to the skin and making it flexible so that it moves along with the skin. Organic and textile optoelectronics may be used to create innovative oximetry sensors in light of these new research avenues.

#### 5.1.5. Blood Pressure

Hypertension and cardiovascular disease are more likely to develop when the body’s reaction to acute and chronic stress is heightened. Disruption in the homeostatic balance may also be characterized as stress. Neuroendocrine and autonomic stress systems must be activated and regulated to restore and maintain homeostasis. In the context of the circulatory system, blood pressure (BP) refers to the force exerted by a person’s blood against the walls of their capillaries [103]. Two measurements are taken: the diastolic and the systolic. BP waves are measured using a pressure-sensitive device. It is possible to utilize a piezo-electric or a compressible dielectric in conjunction with these sensing elements to create capacitance changes or voltages across the circuit. The capacitive components may be amplified using auxiliary equipment or a “monolithically” integrated gadget on thin-film transistors. Piezo-electric elements are also used to create strain-sensitive gadgets. A voltage signal is induced along with a gadget whenever a piezo-electric element is stressed. In addition to the detector itself, the architecture that houses a pulse pressure gauge is critical [104]. Amplification is required in such setups because the baseline pressure is much greater than the pulse pressure. To offer amplification, the amplifier is integrated directly into the detector. PPG monitoring is also utilized for the monitoring of blood BP [86]. When employing the capacitance pressure gauge for carotid arteries, the optical absorption maxima (because of the large amount of blood) are observed. Lee et al. [105] developed an approach for predicting BP. Non-pressurized pulse waves from the wrist-worn pulsimeter with the Hall sensor were used in the study. The magnetism of a “permanent magnet” may be detected via a newly created instrument. Using two silicone-coated “micro-electromechanical” (MEMS) pressure detectors, Stupin et al. [106] demonstrated a noninvasive method of detecting BP waveforms via contact with skin. BP waves are concurrently measured by placing detectors at two places on the body. PWV may be calculated by determining the delayed time between the two pulse waves. The ability to quantify pulse pressure has been shown in a large number of studies, but no studies have been able to show a connection between the obtained signals and blood pressure levels. They identified non-linear associations between systolic BP and detector outputs for individuals who were able to measure BP.

#### 5.1.6. Electrodermal Activity

A variety of physiological and psychological responses are triggered by neurocognitive stress in the body. Stress is associated with an increase in heart rate, salivary cortisol, and skin conductance levels. Other kinds of stress, such as emotional, cognitive, and motivational, may also be found [107]. The “electrodermal activity” measurement (EDA) (galvanic skin response or skin conductance) is a way to determine the flow and distribution of electrical energy in the skin. The skin undergoes modifications when it receives particular brain impulses [14]. Mental, emotional, and physical exhaustion are all possible causes. Measurement of EDA may be done by applying a mild electrical current between two electrodes positioned adjacent to each other on the surface of the skin. A nerve system’s response to anxiety, involvement, enthusiasm, or any other emotion may be captured by an EDA detector. When an individual is anxious, their sensitivity increases because of an increase in the amount of moisture on the surface of their skin, according to Christopoulos et al. in 2019 [108]. When someone is less anxious, their skin conductance decreases. It is commonly employed in the detection of stress and emotions [109]. Table 4 depicts the sensor availability.

### 5.2. Behavioral Stress Assessment and Sensors

Effectively managing behavioral investigations has highlighted the importance of behavioral stress in initiating significant cardiovascular problems such as myocardial injury and infarction, arrhythmia, and sudden cardiac mortality [110]. Sensors for activity tracking are described in detail in the upcoming section. For example, the wrist may be utilized to detect various activity data such as movement and gesturing. Below are several various behavioral markers that might aid with stress evaluation.

#### 5.2.1. Speech Pattern

When an individual is stressed or not, their speech styles might be significantly varied. Whenever people are stressed, their vocal behaviors may shift in a variety of ways, including tone, attitude, and talking tempo, as well as the terms they use in their speech [111,112].

#### 5.2.2. Facial Expression

Natural routines, including facial gestures, express an individual’s cognitive condition and indicate the feeling he or she is feeling. Many studies are using face electromyography (EMG) to record those tiny indicators and connect them with stressful conditions or identification of images based on facial expressions [113,114].

#### 5.2.3. Sleep Pattern

Excellent quality bedtime is crucial for a healthy body, and obtaining enough sleep in today’s world has become a difficult challenge [115]. In the process of premenstrual syndrome (PMS) in teenagers, several studies intended to investigate the impacts of sleeping habits, anxiety, and mindset, as well as behavior that leads to a decrease in exposure to endocrine-disrupting chemicals (EDCs) [116]. Pupils have been more distressed by academic problems than social aspects, according to Gandhi et al. (2021) [115], which resulted in a considerable shift in sleeping habits.

#### 5.2.4. Keyboard and Mouse Dynamics

The majority of individuals presently utilize computers in a routine. The manner individuals utilize things might offer information regarding their current state of mind or sentiments. The speed at which a person moves the mouse or types gives valuable signals about their mental state. Excessive force while pounding a keyboard or hitting a keyboard is unquestionably a symptom of a troubled mind [117,118].

#### 5.2.5. Mobile Phone Usage

**Figure 4:**
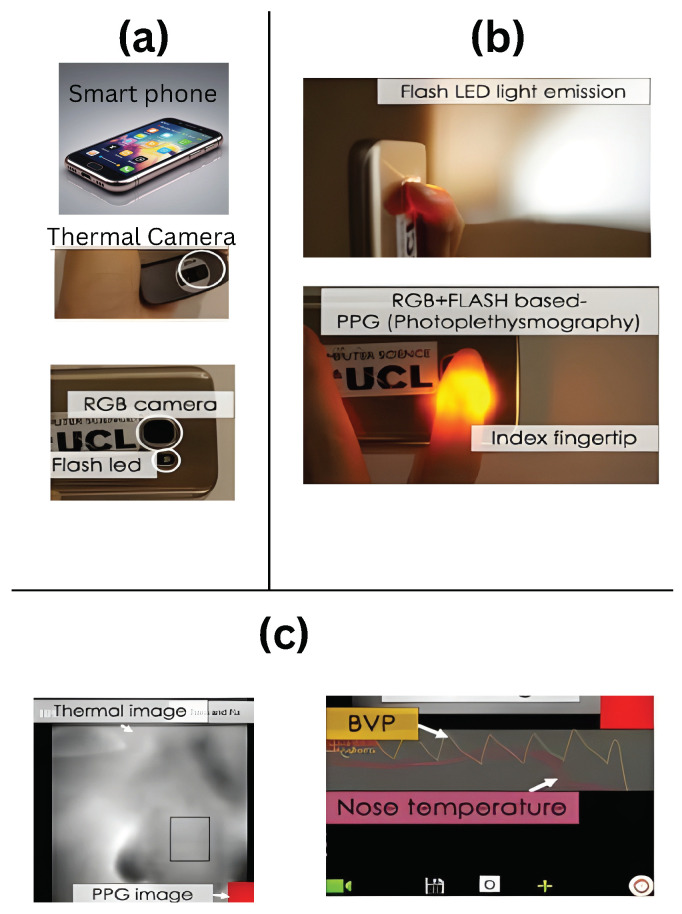
Smartphone RGB and thermal cameras may be used to monitor a person’s physiological state: (**a**) smartphone with a thermal camera, (**b**) flashlight emitting diode emission, and (**c**) designed software interface [119].

**Figure 5:**
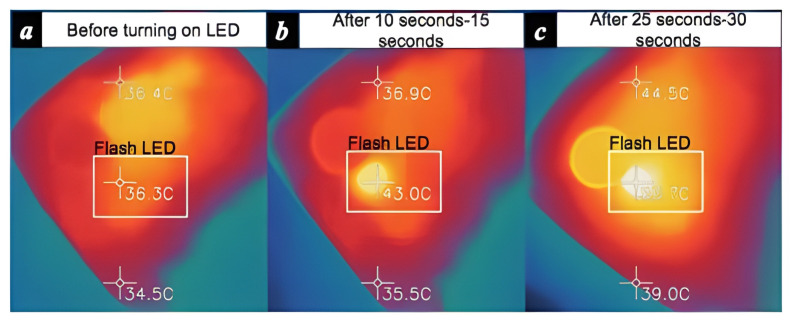
Infrared thermal imaging of a smartphone’s back flashlight emitted diode: (**a**) earlier switching on the LED, (**b**) next 10–15 s, and (**c**) later 25–30 s [120].

An individual who is stressed can fight it or flee from it. Utilizing a mobile device could be a simple approach to concentrating on someone’s tension. Figure 4 and Figure 5 show that Smartphone RGB and thermal cameras may be used to monitor a person’s physiological state and infrared thermal imaging of a smartphone’s back flashlight emitted diode, respectively.

The tension may appear to melt away briefly by diverting oneself with different capabilities incorporated into a cellphone. Technology habits may be an indication of tension, which would be a typical indication in those who are stressed [121]. According to one research by Sano and Picard [119], there is a link between smartphone usage and anxiety. An inexpensive thermal camera may be attached to a smartphone to improve signal quality and identify stress reactions quickly, according to a study by Cho et al., (2019) [120].

#### 5.2.6. Motion and Gestures

A stressed individual’s physical behavior, such as jaw gripping, persistent hand scratching, or especially posture alterations when sitting, are all symptoms of their mental condition. For stress identification, a variety of behavioral indicators are included [122]. The accelerometer is perhaps the most popular sensor for sensing the movement of the individual. For one or even more directions, it determines the rate of velocity. Determining acceleration may be done using a switching frequency that monitors signal width and frequency. Signals with switching frequency corresponding to acceleration emerge from the system’s re-responses [123]. It is possible to identify various individual hand and body motions using a triaxial acceleration sensor and gyroscope sensors in cooperation [124], whereas RGB-D detectors, in conjunction with a triaxial acceleration detector, can identify up to 20 distinct individual actions [125]. Using gyroscope sensing devices, it is possible to tell how the body’s various portions are rotating. They track movement by detecting body movement and rotational acceleration along one or even more dimensions, whereas magnetic detectors are effective in establishing alignment concerning Earth’s geography. Accelerometers are often used in conjunction with gyroscopes. Musculoskeletal motions and actions are detected using “surface electromyography” (sEMG), which measures impulses emitted by neurons at the interface of the tissue. Such detectors can detect wrist movements. Gestures may be recognized using sMMG, which employs piezo-electric pressure detectors. To determine how far a finger is flexed, an array of piezo-electric touch detectors may be placed on the wrist. In research, wrist-based wearable sensor gadgets have been used to measure the quality of sleep [126]. Gyroscopes, accelerometers, and magnetometers, which each have distinct strengths and weaknesses, are often found in a single gadget to maximize efficiency. When it comes to measuring angles, for example, the gyroscope responds quickly and is more accurate than the magnetometer, although the latter is more accurate over time [44].

#### 5.2.7. Body Acceleration

It was found that an accelerometer could measure accelerations as small as 2 g, according to Malhi et al. (2010) [127]. Comparing current body acceleration or motion with prerecorded data can lead to identifying body stress levels. To estimate the acceleration, duty cycles may be used to assess pulse width and duration. The switching frequency of the digital signals produced is directly related to the rate of movement.

#### 5.2.8. Proximity Detection

A wide range of proximity prevention technologies have been designed employing capabilities like ultrasonic sensors, wireless communications monitoring, and electromagnetic fields, as well as radar systems, sonars, and GPS to avoid collisions. To avoid fatalities induced by machinery conflicts, these technologies are most often utilized in construction sites. Awolusi et al. [44] said that Radio Frequency Identification (RFID) is the widely utilized method for proximity detection. To determine the state of people and things, it uses electromagnetic pulses and signals to send information and undertake wireless information extraction and preservation [128]. Because of their low sensitivity to ambient disturbance, ultrasonic instruments have a distinct benefit, but they also carry the risk of reacting incorrectly to loud sounds. GPS-based systems also have the benefit of being simple to use and accurate. GPS-based devices have an edge over other devices, like ultrasonic, radio waves, and laser tracking, in terms of measurements like location, velocity, and alignment [129].

#### 5.2.9. Strain

Strain detectors are devices that monitor changes in the pressure inside the individual body. These detectors are typically tiny metallic foils or semiconductors, and they may be applied to numerous body areas, allowing the user to monitor their strain fluctuations. Comparing the present information from the strain detector with prerecorded data can lead to the identification of the stress level. Wrist-worn strain detectors may also be used to monitor and track wrist mobility, making them valuable in certain occupations (e.g., tennis players) [130]. In [131], A piezo-electric device is the most popular strain–pressure detector type, followed by a capacitive sensor. In terms of use, these two are very regularly employed. Piezo-electric sensors detect electrical charges created by ambient factors, such as vibrations or shocks in the environment (i.e., pressure, strain). Non-centrosymmetric crystal structures are required to induce rapid piezoelectricity. Nevertheless, due to their significant lead toxicities and expensive production costs, polymer-based piezo-electric elements (e.g., PVDF) are also employed [131]. Top and bottom electrodes in the form of flexible capacitive sensors are often made of gold sheets, which make flexible capacitive gadgets. However, carbon nanostructures and silver nanotubes are also being used in research [132]. Table 5 shows the sensors available for behavioral assessment along with their capabilities to identify distress, stress, and sleep quality.

**Figure 6:**
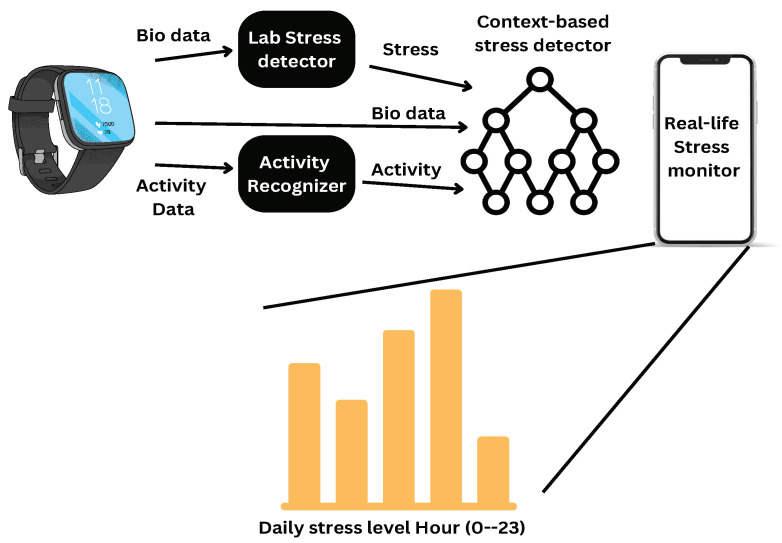
Determining stress using a context-based approach in open situations.

Stress detection using wristbands and cell phones is shown in Figure 6. Stress in real life may be distinguished from a variety of scenarios that elicit physiological responses comparable to those caused by context-based stress detectors. Statistics are applied to the four experimental stress detector outcomes, the preceding context-based detector outcome, and the 20th percentile of each sensor’s data set as additional features (HR, BVP, IBI, ST, and EDA signal). The context-based classifier employed the 20th percentile to obtain some information from each sensor [14].

### 5.3. Questionnaire and Survey

In mental and emotional studies, questionnaires and polls are generally commonly employed to measure emotional states. Individuals may reveal the innermost problems or stresses in their thoughts by interviewing questionnaires that help to discover a certain cognitive attitude. Sometimes, the individual may not be aware of the cause of their anxiety, therefore necessitating the use of questionnaires or psychological consultations. Panicker et al. (2019) [1] provide a thorough examination of the essential aspects of cognitive anxiety monitoring methods: Acquisition of physiological parameters, the function of pattern recognition in Sentimental Analysis and Anxiety Monitoring devices, multiple assessment methods, problems, and implementations. Subjects’ responses to questionnaires and interviews may be used to compile behavioral stress ratings. Immediate disclosure of information and day reports are two methods for collecting information from people in their everyday lives. Foster and Bradley [133] found that people tend to ignore sentimental maxima within 24 h. Whenever accurate assessment is needed, do not ask subjects questions towards the end of the day. As a solution, one might have the individual immediately disclose any stressful situations that have occurred to them. There is an issue with this approach since individuals may neglect to document occurrences. Combining these strategies may help improve the quality of reports, as a consequence. In laboratories and everyday life stress trials, interviews, and questionnaires such as the “Perceived Stress Scale” (PSS), the “Stress Self-Rating Scale” (SSRS), NASA-TLX, and the “Self-Assessment Manikin” (SAM) are often employed.

#### 5.3.1. NASA Task Load Index

**Figure 7:**
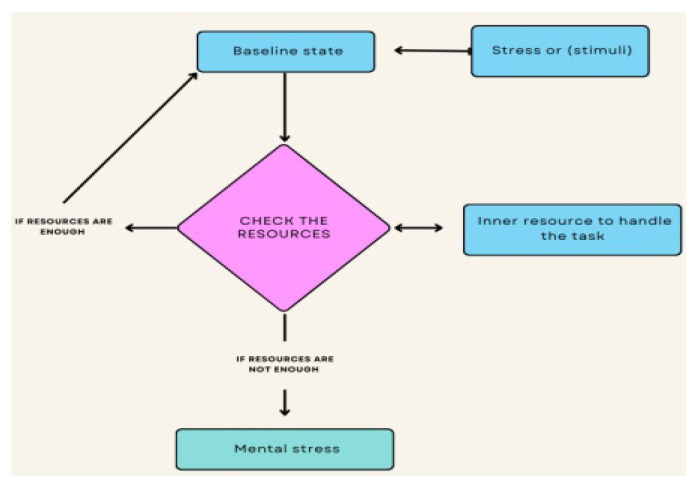
Stress handling process as per human behavior.

To gauge how much work people think they are doing, NASA created the “NASA Task Load Index” (NASA-TLX) [134,135]. Performance objectives in particular operating situations are often assessed using this instrument. Since its inception in aviation, it has found use in a variety of other fields, including personal aspects studies. When one or even more subjects are engaged in an activity, a six-dimensional index is generated. Effectiveness, persistence, and dissatisfaction are measured on these measures. NASA-TLX is divided into two sections. In the first section, the respondent is asked to rate each scale from 0 to 100. Six questions make up this section. The second portion of the inquiry is meant to provide a numerical value to each rung of the grading system. For every level, the participant is instructed to do a pairwise assessment. There are 15 questions in the second section. Figure 7 shows a schematic flow of the NASA Task load index.

Table 6 provides a comparative analysis of the studies reviewed in this SR. In the majority of the investigations that measured anxiety, HR and HRV were the primary indicators of anxiety.

#### 5.3.2. Bridging Technology and User Experience

Technological Landscape: Smart wearables are evolving rapidly to address mental health challenges by leveraging advanced technological innovations. These devices offer continuous physiological monitoring by measuring key stress indicators such as heart rate variability (HRV), electrodermal activity (EDA), respiratory patterns, and skin temperature. These capabilities form the foundation for real-time stress analysis and intervention.

User-Centered Intervention Strategies: Wearable technologies provide a structured approach to stress management through three primary intervention strategies. Real-time stress detection enables immediate recognition of physiological signals, delivering instant biofeedback and personalized stress intervention recommendations. This immediacy facilitates proactive responses to stress events, fostering self-awareness and immediate coping mechanisms. Support for self-regulation is a central feature, with wearables offering guided breathing techniques, mindfulness prompts, and stress awareness notifications to encourage healthier emotional management. Additionally, long-term mental wellness tracking empowers users by analyzing health trends over time, generating personalized insights that complement traditional therapy and medical interventions [136,137,138].

Practical Impact and Research Findings: The practical benefits of wearable technologies in mental health management are increasingly supported by empirical research. For instance, a randomized workplace intervention study revealed measurable mental health improvements among participants, with a 15.8% reduction in negative stress occurrences, a 13% decrease in distressing symptoms, and a 28.2% decline in anxiety days. Such findings emphasize the potential of wearables to serve as transformative tools in mental health management [139,140].

User Experience Considerations: Despite their potential, wearable technologies can introduce challenges, particularly for users prone to anxiety. Continuous monitoring of health metrics may induce stress, create information overload, and occasionally contribute to obsessive health-related behaviors [141]. These challenges underline the importance of developing user-centered design principles that minimize unintended consequences while enhancing user satisfaction. Technological Innovations: Emerging wearable devices showcase the intersection of advanced technology and innovative design. For example, Fitbit Sense 2 offers continuous EDA monitoring, while the Happy Ring combines mental and physical health metrics for holistic assessments. The Apple Mindfulness App integrates reflective wellness tools, bridging technological functionalities with mental health support systems. Ongoing research is critical for refining wearable technologies to achieve more accurate and meaningful health outcomes. Future objectives include enhancing the precision of stress detection algorithms, developing nuanced intervention strategies tailored to individual needs, and creating user-friendly, non-intrusive monitoring systems. Addressing these research priorities will ensure that wearable devices continue to evolve in alignment with the diverse requirements of their users [138]. Modern wearable technologies represent a transformative advance in mental health management, offering personalized and continuous support to users. The challenge lies in balancing cutting-edge innovations with human-centered design principles to create intuitive, supportive, and empowering devices. Wearables that seamlessly integrate advanced sensing capabilities with thoughtful user experiences have the potential to redefine mental wellness practices, providing users with tools to navigate their mental health journeys effectively [136].

## 6. Limitations and Future Scope

In this section, we have mentioned the drawbacks or limitations found in the literature, along with the future directions. The paramount contributions of some of the studies are also mentioned.

- Heart Rate Variability over other physiological measures

Numerous cardiovascular disorders, like myocardial ischemia, heart attack, sudden cardiac death, hypertension, dementia, older adults with cortisol and obesity, along with mental wellness issues such as stress and anxiety in various cases [142,143,144,145,146,147,148,149,150,151,152,153], have been linked to abnormal HRV measures. An Apple Watch-based study by Hernando et al. (2018) [80] found that HRV readings are better compared to HR alone in the detection of anxiety, with most available on-the-market gadgets relying on the mean HR, which would be tightly monitored by the autonomous nerve mechanism and can also modify under specific pathological conditions and pathophysiologic conditions. In addition, HRV measures not only HR but will show modified autonomic functioning in these settings. It was shown that the “Polar V800” HR detector, which detects HRV, was highly correlated with ECG HRV underneath a variety of stresses in the research by Huang et al. (2021) [154]. To a similar degree to an ECG, such portable gadgets are capable of detecting high levels of anxiety, according to this research. Portable sensors may be more precise and practical than hormonal and neurotransmitter analyses when it comes to monitoring the body’s response to stress, according to a study published in the Journal of Clinical Endocrinology and Metabolism. While many devices still utilize average HR, HRV characteristics are the most trustworthy in identifying anxiety. In addition to HRV, many studies looked at EEG for anxiety identification. Anxiety has been detected in the past using an asymmetrical assessment of EEG frequency-band strengths recorded in the prefrontal brain. SVM (Gaussian Radial Basis Function) was shown to be better efficient in detecting anxiety in the EEG relative to the ECG alone (92.70 percent), therefore proving that EEG and HRV are more useful for anxiety identification when used together. Although EEG was shown to be more responsive to anxiety identification in this research, its potential to be integrated into a small and aesthetically attractive wearable sensor is yet restricted. According to Pakhomov et al. (2020) [155], who utilized Fitbit to measure heart rate at rest and after response to anxiety, the HR increased by a mean of nine pulses every minute after the stress response. Lucas et al. (2019) [156] and Pakhomov et al. (2020) [155] found that HR could be beneficial in identifying anxiety. Although both investigations were constrained to the tender age of their participants, hence the effects of comorbidity on HR and, consequently, anxiety identification may not be indicative of the overall community. Furthermore, it is well-established that the maximal heart rate (HR) falls linearly with age, with sleep and stress levels shifting significantly throughout a person’s lifetime. Wearable sensors may need to be adjusted to account for the physiological differences between men and women, but it looks like HRV measures may render average HR identification unnecessary.

A separate investigation by Seoane et al. (2014) [97] found that multi-parametric evaluation encompassing GSR, thermometer, breathing rates, and ECG through a model wearing clothing had a better performance of detection anxiety over EDA. Inconsistencies in GSR and body temperatures were nearly twice as prevalent as in ECG and respiration rate when using the wearable system to monitor distress. Additionally, gadgets like these and feedback systems for lowering anxiety may help alleviate the load that anxiety places on individuals in their daily lives.

- Inconsistencies with EDA

Skin conductivity rises when people are anxious, which was discovered to be beneficial in detecting “neurocognitive” stress in the current SR, which revealed a gadget not recognized with the aforementioned search criteria. Instruments called “shimmer sensors” are worn on the body to assess anxiety levels, and they employ EDA to identify anxiety in 86 percent of individuals in a single trial. Employing the wrist-worn ADI VSM, another research was identified that evaluated EDA to identify the amount of pre-surgery stress. Anusha et al. (2019) [109] found that gadgets that use EDA information to identify anxiety are vulnerable to movement artifacts. They also found that pressure variations on EDA electrodes caused by changes in the tightening of the accessory and movements of the hands and wrists can disrupt the information significantly, leading to untrue observations, which is believed to be a significant concern with EDA.

- Differentiation of stress with laziness

Furthermore, evidence that might assist in differentiating stress from laziness, as well as favorable and unfavorable impressions, should be considered in the future. Examples include the use of an accelerometer and gyroscope as a marker for anxiety or sadness or the use of location information for a variety of purposes other than the ones we investigated in this study. When combining phone and ambient monitoring information, like movement detectors and keypad usage statistics, it may be worthwhile. This research might be furthered by gathering long-term data on a larger number of people and studying the differences between them as well as various sorts of stresses. A wide range of studies has shown that stress levels vary widely among persons. In terms of anxiety, self-assessment is widely accepted as a reliable tool. However, this method needs individuals to continually monitor and analyze their situations, which might raise their mental burdens and their overall stress levels. Sensor-based anxiety identification does not necessitate any endeavor from the participants and thus may enable autonomous identification of specific stressful events and individualized mentorship to assist and cope with problems. For example, it could be helpful to know whether a person’s daily stresses are cognitive-emotional, societal, physical, or ecological and give the proper level of assistance. People’s dealing mechanisms and reactions to anxiety are influenced by their personality traits and taught dealing strategies, but chronic stress is the most detrimental kind since it happens when stressors continue and endure for an extended period. Anxiety data will be supplemented in the future by data on possible everyday life stresses [157,158].

- Artifacts identification from limitless movements

Unhindered development of individuals and inappropriately worn gadgets are other critical difficulties. In research center conditions, developments and exercises are restricted and obliged. Analysts obtain the opportunity to intercede with subjects to wear gadgets appropriately. Nonetheless, in regular routines, developments are unlimited. Individuals even will quite often make more than each movement in turn which makes the identification cycle more complicated. This could lessen the exhibition of stress location frameworks and increment movement antiques. Movement acknowledgment plans might be utilized with stress-identification frameworks to improve exactness. Recognition of activities should be used with stress recognition systems to improve efficiency [159].

- Differentiation of stress from other physical activities

Acceleration has been proven to be the most successful signal to differentiate physical activities and stressful and relaxed states. Apart from the acceleration, it was discovered that the second most useful physiological factor is heart activity. HRV alone has a maximum of 70 percent accuracy for the differentiation of physical activity and stress. Integrating these two signals will give a system that is robust for differentiating the signals. The benefaction of this exploration will be helpful to both the scholarly world and industry, and it very well may be utilized to further develop the algorithms running on smart bands. This study could be utilized for a more profound comprehension of physiological responses of pushed and dynamic states and it will direct the researchers in the evolution of more sturdy algorithms for detecting stress in the daily lives in a continuous manner. Moreover, the researcher has used traditional machine-learning algorithms in his work though deep-learning algorithms can likewise be taken advantage of for this. In any case, the physiological signals were not investigated when mental stress happens during a physically active state [160]. Table 6 provides a comparative overview of technologies reviewed, focusing on their respective sensors, methodologies, and applications in psychological, behavioral, and environmental assessments.

### 6.1. Current Challenges in Wearable Mental Health Technologies and Future Directions

Smart wearable devices for mental health monitoring offer significant potential, yet their widespread adoption and efficacy are hindered by various challenges.

The first major limitation lies in data interpretation. Despite advancements in sensor technologies, many users face difficulties in understanding the raw physiological data generated by wearables. Even individuals with adequate digital literacy often find it challenging to derive meaningful insights about their mental health from these metrics, such as heart rate variability or skin conductance. This gap highlights the urgent need for more intuitive interfaces and user-friendly systems capable of delivering actionable health insights [161].

Another significant barrier involves privacy and security concerns. Nearly 40% of users report apprehensions about how their health data are stored and utilized, which raises fears about potential misuse or unauthorized access. These concerns directly impact user engagement, as many individuals hesitate to fully integrate wearable devices into their health routines without robust assurances regarding data protection [162,163].

In addition to technological and privacy-related barriers, user expectations for wearable technologies often exceed the current capabilities of these devices. Modern users expect wearables to offer advanced functionalities that move beyond basic tracking. Desired features include highly accurate blood pressure monitoring, comprehensive glucose tracking, and nuanced emotional state assessments. When these expectations remain unmet, users experience dissatisfaction and distrust in the technology, which further impedes adoption [162,163].

The public health implications of wearable technologies for mental health highlight additional challenges. Research efforts have revealed several key gaps, including a limited understanding of how users engage with and perceive wearable devices on an individual level. Existing studies often overlook user satisfaction factors and fail to establish comprehensive frameworks for integrating wearable-generated data into holistic health management practices. Addressing these gaps would require concerted research into the usability, personalization, and integration of wearables into broader healthcare systems [164,165].

Wearable mental health technologies also face critical methodological challenges. Research in this area often lacks controlled studies with validated measurement protocols, leaving gaps in the reliability and accuracy of device outputs. Furthermore, there is a pressing need for sophisticated algorithms capable of delivering real-time mental health feedback, which has yet to be adequately developed or tested. Existing methodologies frequently fall short when applied to real-world scenarios, emphasizing the necessity for rigorous testing in diverse and practical environments [164].

To advance the field, future research must prioritize enhancing user experience through intuitive interfaces, personalized mental health insights, and innovative engagement strategies. Concurrently, researchers and developers need to establish robust ethical frameworks that address data privacy concerns. By implementing stringent data protection mechanisms, creating transparent policies for data use, and enabling users to control data-sharing decisions, these technologies can gain broader acceptance and trust [165].

Incorporating more advanced sensing technologies remains another critical direction for future research. Efforts should focus on improving multi-parameter stress detection methods, leveraging machine learning to enhance emotional state predictions, and developing noninvasive, continuous monitoring systems to maximize user comfort and data accuracy. However, despite significant advancements, wearable mental health technologies still face substantial adoption barriers, including complex interfaces, difficulties in data interpretation, and ongoing privacy concerns. High device costs, coupled with inconsistent accuracy, also pose challenges to equitable access and reliability [166].

The successful development and widespread implementation of wearable technologies for mental health hinges on overcoming these multifaceted challenges. The integration of innovative design, ethical data practices, and advanced technology requires collaborative efforts between technologists, healthcare providers, and end users. By addressing these critical gaps, wearable technologies can achieve their full potential in improving mental health outcomes and contributing to overall wellbeing.

#### 6.1.1. AI’s Transformative Role in Wearable Technologies

Artificial Intelligence (AI) is reshaping wearable technologies by embedding advanced computational capabilities that redefine how health data are processed and utilized. Through strategic AI integration, wearables are evolving from simple monitoring devices into proactive, intelligent health management systems. Core AI Integration Strategies AI enhances wearable technology by leveraging advanced machine-learning algorithms for processing complex sensor data. These algorithms allow real-time physiological signal analysis, transforming raw data into actionable, personalized health insights. The deployment of AI in wearable devices ensures a high degree of precision, adaptability, and user-centric functionality.

#### 6.1.2. Machine-Learning Applications

Various machine-learning techniques play pivotal roles in wearable functionality. Convolutional Neural Networks (CNNs) are employed for feature extraction and pattern recognition, especially in visual and spatial data interpretation. Recurrent Neural Networks (RNNs) and Long Short-Term Memory Networks (LSTM) facilitate the processing of sequential and temporal data, ensuring accurate trend analysis and predictions. Hybrid deep-learning models further enhance these capabilities by integrating the strengths of diverse approaches to improve overall system efficiency and accuracy [167].

#### 6.1.3. Advanced Technological Innovations

AI-powered wearables boast capabilities such as comprehensive health metric tracking and predictive monitoring of potential health conditions. They enable continuous physiological assessment and provide tailored intervention strategies to users. These innovations empower users to make informed decisions regarding their health, fostering better long-term outcomes [167,168].

#### 6.1.4. Practical Implementation Areas

AI applications in wearable technologies span diverse domains, underscoring their versatility. In healthcare, AI enables early diagnostics and effective chronic disease management. Fitness and performance tracking is enhanced through personalized insights, while mental health monitoring is revolutionized by predictive stress detection and tailored mindfulness recommendations. Such multifaceted functionality positions AI-powered wearables as integral tools in modern health management [169].

#### 6.1.5. Emerging Technological Trends

Breakthrough advancements continue to emerge in AI-powered wearable technology. Devices now monitor blood glucose levels non-invasively, analyze sleep patterns with high precision, detect stress through multimodal sensing, and evaluate metabolic functions dynamically. Personalized nutrition recommendations are another innovative feature, utilizing AI algorithms to provide actionable dietary advice based on individual metabolic data [167,168,169]. Artificial Intelligence is transforming wearable technologies by adding layers of intelligence that empower individuals with personalized health management capabilities. These devices are no longer passive data collectors but proactive partners in promoting physiological and psychological wellbeing. AI’s role ensures a future where wearables integrate seamlessly into everyday life, offering insights that foster healthier, more informed decision-making [167,168,169].

Smart Contact Lenses: Transforming Health Monitoring Smart contact lenses represent a groundbreaking technological advancement, offering a wide range of innovative features for health monitoring. These devices, initially designed for vision correction, are evolving to serve as sophisticated health monitoring tools, promising to revolutionize the way healthcare is delivered. Smart contact lenses are capable of noninvasive glucose tracking by analyzing tear fluid, allowing individuals to monitor their glucose levels without the need for traditional blood sampling. This feature includes real-time wireless data transmission to smartphones, offering convenient and continuous monitoring. Such advancements hold the potential to significantly change the landscape of diabetes management, providing users with timely insights into their glucose levels and fostering proactive healthcare interventions. The lenses also facilitate continuous intraocular pressure (IOP) monitoring, an essential tool for glaucoma detection. Advanced sensors integrated into the lenses track variations in eye pressure, allowing for early and noninvasive screening of glaucoma, a critical factor in preserving eyesight. The technology is FDA-approved for this purpose, positioning smart contact lenses as a vital tool for early diagnosis and ongoing monitoring of eye health. Beyond monitoring, smart contact lenses also hold promise for delivering medical treatments directly to the eyes. They are designed to release antihistamines for allergy relief and deliver targeted medications for specific eye conditions. This approach promises to reduce systemic side effects by providing localized, controlled treatment, enhancing the effectiveness of medication while minimizing unwanted side effects. Technological Innovations: Smart contact lenses boast several advanced technological innovations. Key features include wireless data transmission for seamless connectivity with smartphones and healthcare devices, embedded microelectronics that make the lens capable of sensing and transmitting data, and biocompatible hydrogel materials that ensure comfort and long-term use. Additionally, the lenses may incorporate temperature-adaptive sensing mechanisms to provide real-time adjustments based on environmental conditions, further enhancing the accuracy of the measurements. Future Potential of smart contact lenses: The future of smart contact lenses appears promising, as they are poised to revolutionize healthcare in numerous ways. Their ability to offer continuous, noninvasive health monitoring positions them as a potential cornerstone of personalized medical care, enabling tailored treatment plans. The lenses could provide real-time, actionable data for physicians and patients, driving earlier disease detection and more precise interventions. As technology advances, the range of applications may expand beyond current uses, unlocking new potential for disease prevention, diagnostics, and health management. Smart contact lenses are transforming the traditional concept of vision correction by integrating groundbreaking health monitoring capabilities. From glucose tracking to glaucoma screening and medication delivery, these lenses are becoming versatile tools with wide-reaching implications in medical diagnostics and personalized treatment. As this technology continues to evolve, it is clear that smart contact lenses could play a pivotal role in improving healthcare delivery and patient outcomes, making a profound impact on both individual health management and public health at large [170,171].

## 7. Conclusions

In this SR, the major emphasis was on wrist-wearable gadgets, including the detectors utilized in such gadgets and the techniques for intelligently interpreting knowledge acquired from those detectors. Data visualization techniques and prominent commercialized wrist-worn gadgets have also been discussed. Healthcare tracking and athletics monitoring are two popular uses, although other particular situations were also found. The concept of wrist-worn instruments that employ a particular collection of detectors or novel types of detectors that are not usually seen in gadgets on the market was recognized by this SR. There are a variety of detectors that have never been used in wrist-worn devices, but maybe because of their architecture and design. No taxonomy for wrist-wearable gadgets has been found in the literature study. RQ1 helped us to discover the essential components and operation of wrist-wearable gadgets. As a result, detectors built into wrist-worn gadgets that monitor physiological and interaction data, along with ecological variables, have been thoroughly examined. Non-invasively, such gadgets assess the relevant physiologic criteria: HR, skin temperature, BP, blood oxygen levels, sugar levels, BVP, EDA, and electrolytes. Moving around and using movements may all be used to gather data about activities. As a final point, wrist-worn devices can measure atmospheric temperature, elevation, and G-force in addition to a host of other climate data, such as illumination and audio levels. According to the investigation, commercialized wrist-worn noninvasive gadgets most often assess HR. When it comes to commercialized alternatives, the most common method of determining HR is via the use of a PPG detector. The use of PPG in wrist-worn gadgets and the acquisition of precise readings is the subject of several deployments and investigations. The study also found that oxygen saturation levels and sugar levels are assessed in certain investigations and designs but not in commercially marketed gadgets. Even though there are many detectors, the HR sensor and movement sensors, such as accelerometers and position sensors, are the most prevalent in wrist-worn gadgets, whereas other detectors are employed only intermittently. Complimentary detectors that can recognize a wider variety of actions are essential when designing a sensor-based gadget. There is a very thin line between commercialized items and experimental models, according to our findings (RQ2). For tracking wellness and activity, commercialized solutions rely primarily on older, low-cost, standardized, and well-established sensors. While commercially available detectors have yet to fully develop, experimental models are presenting novel detectors with novel functions. Noninvasive sugar level sensors have recently been offered as a proof of concept, for instance. According to RQ3, we were tasked with examining existing investigations using wrist-worn sensors to measure anxiety and identify study gaps. Optimization algorithms that operate on or with wrist-worn gadgets were thoroughly examined for this report. The most important point from this is that the gadget itself does not have any inbuilt advanced technologies. The vast majority of studies involving the utilization of detector information from wrist-worn gadgets in information assessment were completed offline after the information collection phase. Developments in noninvasive wrist-worn technologies have been discussed in this SR. Analyzing existing technology evolution, data visualization approaches and smart information processing were all part of the review’s goal. It also compared many prominent commercialized wrist-wearable technologies. The taxonomy of sensing gadgets, functions, and techniques used in wrist-wearable gadgets was one of the conclusions of the assessment due to the lack of a well-established taxonomy. Because of the growing popularity of specialized computational chips, it would then be easier to assess information on the machine itself in the coming years. There is a necessity for further distance measuring technologies studies to scientifically evaluate sleep disturbances, lower socializing and physiological activities, as well as mood changes, prosody, and cognitive function, all of which are helpful sadness markers. Instruments in movement trackers and cellphones are used in the passive remote assessment technique, which autonomously collects statistics to determine whether the user is displaying communications and behavioral habits consistent with a depressing event. Wearable technology’s capacity to offer users timely wellness details will continue to improve. As a result, further research into the validity of wearable technology and the collection of information on intelligent machines for interpreting will be very advantageous.

## Figures and Tables

**Table 1:** Search strategy keywords.

	Keywords
1	What is stress?
2	Stress detection using smartphones and wearable sensors
3	Monitoring stress with a wrist device using context
4	Prediction of stress levels
5	The Stress of Life
6	Emotional distress monitoring methods
7	Machine-learning techniques for stress detection
8	Stress Detection in the working environment
9	Behavioral stress detection
10	Stress wearable devices
11	Health monitoring system for sports person
12	Detecting negative stress
13	Emotional stress-monitoring
14	Effect of sleep on health and stress
15	Stress-induced brain activities

**Table 2:** Exclusion and Inclusion principles for SR.

Inclusion Criteria	Exclusion Criteria
I1: The paper should be peer-reviewed	E1: Papers which do not focus on the Body Stress-related study
I2: The Paper should be in English language	E2: Grey literature
I3: No time frame limit of publication	E3: Duplicate research and publications
I4: Papers should be a published research or full-article publication	E4: Ph.D. theses, working papers, and project deliverables
I5: Standard of paper was blind to impact factor	

**Table 3:** Environmental conditions detecting sensors available in smartphones, smart bands, and smartwatches.

Sensors	Smart Phone	Smart Band	Smart Watch
Accelerometer	Yes	Yes	Yes
Proximity sensor	Yes	Yes	No
Photoconductivity detector	Yes	Yes	Yes
Infrared detector	Yes	Yes	Yes
Thermistor	Yes	Yes	Yes
Pressure or Strain	No	Yes	Yes
Photodetector	Yes	No	Yes
Electrochemical detector	No	Yes	Yes
Gyroscope	Yes	Yes	Yes
Magnetometer	Yes	No	Yes
Surface electromyography	No	Yes	Yes
Ultrasonic detector	No	Yes	No
Radiofrequency detector	Yes	Yes	Yes
GPS	Yes	Yes	Yes
Piezo-electric sensor	No	Yes	Yes
IMU	Yes	Yes	Yes
Motion Detector	Yes	Yes	Yes
EDA	No	Yes	Yes
Inter-beat interval (IBI)	No	Yes	Yes
EDC	No	Yes	Yes
Pulse detector	No	Yes	Yes

**Table 4:** Sensor availability table for Psychological Assessment.

Input Signals	Distress/Illness	Stress/Wellbeing	Sleep Quality
EDA	Yes	Yes	No
IBI	Yes	No	Yes
ECG	Yes	Yes	Yes
PPG	Yes	Yes	No
Temperature	Yes	No	No
Oxygenation	Yes	Yes	No
BP	Yes	No	Yes
BVP	Yes	Yes	No
PWV	Yes	Yes	Yes

**Table 5:** Sensor availability table for Behavioral Assessment.

Input Parameter	Distress/Illness	Stress/Wellbeing	Sleep Quality
Gesture App Usage	Yes	Yes	No
Keyboard and Mouse usage	No	Yes	Yes
Phone usage pattern	Yes	Yes	Yes
Speech pattern	Yes	Yes	Yes
Sleep pattern	Yes	No	Yes
Body Acceleration	No	Yes	Yes
Proximity sensor	No	Yes	Yes
Strain detector	Yes	Yes	No

**Table 6:** Comparison of Technologies Reviewed in Terms of Sensors, Methodologies, Applications, Materials, and Fabrication Methods.

Category	Sensors	Methodologies	Applications	Materials	Fabrication Methods
Psychological Assessment	EDA, IBI, ECG, PPG, Temperature, Oxygenation, BP, BVP, PWV	Signal processing for physiological monitoring	Stress assessment, illness detection, sleep quality analysis	Conductive polymers, carbon nanotubes, flexible substrates (e.g., silicone)	Microfabrication, screen printing, inkjet printing for flexible sensors
Behavioral Assessment	Gesture app usage, keyboard and mouse usage, phone usage patterns, speech patterns, sleep patterns, body acceleration, proximity sensor, strain detector	Behavioral pattern analysis through device interaction	Stress/wellbeing monitoring, sleep analysis, illness detection	Integrated circuit (IC) chips, accelerometers, strain gauges, MEMS (micro-electromechanical systems)	Printed electronics, MEMS fabrication, wafer bonding for compact integration
Environmental Sensors	Accelerometer, proximity sensor, photoconductivity detector, infrared detector, thermistor, pressure/strain, photodetector, gyroscope, GPS, EDA, etc.	Environmental sensing and motion detection	Activity tracking, environmental monitoring	Metal oxides, photodiodes, piezo-electric materials, thermistors, optical fibers	Laser engraving, etching, sputtering for deposition, 3D printing for enclosures

## Data Availability

Not applicable.
